# Brown juice processed from alfalfa green biomass as a source of phytohormones and saponins

**DOI:** 10.1038/s41598-025-03896-7

**Published:** 2025-05-28

**Authors:** Nóra Bákonyi, Döme Barna, Wildan Suhartini, Zoltán Cziáky, Makleit Péter, Tarek Alshaal, Miklós Gábor Fári, Éva Domokos-Szabolcsy

**Affiliations:** 1 Institute of Applied Plant Biology, Faculty of Agricultural and Food Science and Environmental Management, University of Debrecen, Böszörményi Street 138, Debrecen, 4032 Hungary; 2 Food Technology Study Program, Faculty of Industrial Technology, Sumatera Institute of Technology, South Lampung 35365, Bandar Lampung, Indonesia; 3 Agricultural and Molecular Research and Service Institute, University of Nyíregyháza, Nyíregyháza, 4407 Hungary; 4 Soil and Water Department, Faculty of Agriculture, Kafrelsheikh University, Kafr El-Sheikh, Kafrelsheikh, 33516 Egypt

**Keywords:** Alfalfa brown juice, Lacto-fermentation, Plant hormones, Saponins, Medicagenic acid, Plant biotechnology, Screening, Biochemistry, Metabolomics

## Abstract

The valorization of by-products from green biorefineries is critical for environmental sustainability and economic viability. During the extraction of leaf proteins from alfalfa green biomass, significant volumes of brown juice (BJ) are generated. This study assessed the phytohormonal profile and anti-nutritional elements in BJ post-lacto-fermentation, examining its potential as a plant biostimulant. Fresh BJ was lacto-fermented using Pediococcus acidilactici, Lactobacillus paracasei, and Lactobacillus plantarum at 35 °C for 48 h. Phytochemical analysis via HPLC-MS/MS identified 18 saponins in BJ, with only medicagenic acid remaining post-fermentation, whose concentration increased 39-fold. Additionally, 23 plant hormone derivatives were quantified, with significant increases observed in indole-3-acetic acid, gibberellin GA1, jasmonic acid, and abscisic acid, while others like p-coumaric acid, caffeic acid, and ferulic acid decreased. These results demonstrate lacto-fermentation’s ability to stabilize BJ, reduce haemolytic saponins, and enhance phytohormone concentrations, supporting its utility as a sustainable plant growth enhancer.

The isolation of leaf protein through the refinement of green biomasses offers a potential remedy for the current protein scarcity issue^1,2^. Using fresh green biomass as a source of protein is historically reviewed by^3^ Alfalfa (*Medicago sativa* L.) stands out as a widely utilized biomass crop in green biorefineries, primarily due to its abundant protein content suitable for both human and animal consumption^4,5^. Brown juice (BJ), also known as deproteinized plant juice (DPJ) or phytoserum, represents a substantial liquid by-product generated in large quantities during leaf protein production^6^. The proper utilization and integration of BJ into the circular economy are imperative, given the limited knowledge regarding its composition. Recent research has revealed that BJ contains a wealth of nutrients, biologically active compounds, pigments, vitamins, enzymes, minerals, and phytochemicals^4,7^. Consequently, BJ holds potential as a fermentation feedstock for enzymes, biodegradable plastics, amino acids, vitamins, alcohols, and their precursors^7^. Moreover, BJ shows promise as a growth medium for microalgal cultivation and plant nutrition, serving as a biostimulant for enhanced growth and development^4,8^. Alfalfa stands out among plant species for its elevated levels of anti-nutritive elements, specifically belonging to the saponin group^9^, which may not be advantageous and could potentially pose risks to certain livestock. Saponin constitutes the primary anti-nutritional factor in alfalfa^10^, with twenty-four saponins identified in both above- and belowground portions, including mediagenic acid, hederagenin, xanthic acid, soyasapogenol A, soyasapogenol B, soyasapogenol E, and bayogenin glycoside^11,12^. However, the inclusion of 2% alfalfa saponin in pig feed has shown benefits, leading to increased daily weight gain and reduced incidence of diarrhea by enhancing piglets’ digestive physiology^13^. Furthermore, saponin exhibits potential advantages for antimicrobial, fungicidal, nematicidal, insecticidal, and cytotoxic purposes^14^. Given the conflicting effects observed, there is a pressing need and escalating demand for strategies to eliminate anti-nutritional compounds like saponins during product development^15,16^.

Several methods have been employed to reduce anti-nutrient levels, encompassing soaking, various forms of heating such as dielectric and infrared methods, extrusion, bioprocessing, fermentation, irradiation, and non-thermal processing techniques like pulse electric fields and ultrasonic high hydrostatic pressure^17^. Lacto-fermentation is recognized for its capacity to diminish anti-nutritional elements in diverse ingredients, thereby enhancing ingredient quality through significant enhancement in protein biosynthesis, vitamin and amino acid levels, nutrient availability, and macro and microelement content^18^.

Thus, the aims of this study were to: (1) examine the composition of plant hormones in alfalfa-derived BJ, (2) quantify the plant hormones present in BJ, (3) assess the anti-nutritive risks in fresh alfalfa-derived BJ by identifying anti-nutritional components, and (4) elucidate the impact of lacto-fermentation on BJ stability and composition.

## Results and discussion

### Screening of anti-nutritional compounds in alfalfa BJ

**Fig. 1:**
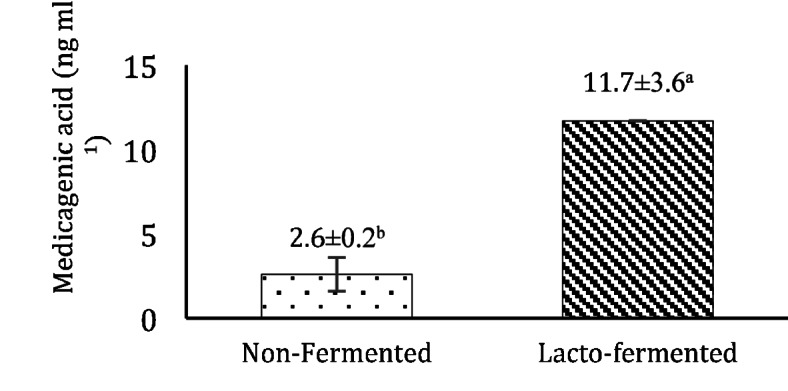
Effect of lacto-fermentation on the concentration of medicagenic acid in alfalfa-derived brown juice (BJ). Sample size (*n* = 3). Different letters following means in each column show significant differences at *p* < 0.05.

Table 1showcased 18 distinct anti-nutritional phytochemicals detected in non-fermented BJ. However, following lacto-fermentation, most of the saponins were eliminated, leaving only the mediagenic acid aglycon detectable. All compounds were identified via negative ionization. Among these, alfalfa-specific azukisaponin II was found in non-fermented alfalfa BJ along with eight unidentified saponin components^19^, previously reported four in their investigation of BJ from alfalfa treated with various selenium forms. However, the remaining four unknown saponins have yet to be documented. Additionally, eight different medicagenic acid derivatives were detected in the non-fermented BJ, along with medinoside E or its isomeric counterpart. Several studies have noted the presence of azukisaponin II, medinoside E or its isomers, and eight distinct medicagenic acids in alfalfa^14,20^, all of which were identifiable in alfalfa-derived BJ. Lacto-fermentation is known to degrade anti-nutritional components in various materials, thereby enhancing feed and food quality by promoting the biosynthesis of proteins, vitamins, amino acids, and nutrient availability^18^. Consequently, lacto-fermentation nearly eliminated all detected saponins in BJ, except for medicagenic acid, rendering it more beneficial for further applications. It should be noted regarding medicagenic acid that different medicagenic acids with sugar tails (glycoside form) identified via screening were eliminated during lacto-fermentation as the different sugars were consumed by bacteria during fermentation, forming aglycon form, increasing the concentration of medicagenic acid is shown in Fig. 1.

### Quantification of anti-nutritive compounds of non-fermented and fermented alfalfa BJ

Medicagenic acid and its derivatives are among the abundant saponins in alfalfa^14^. The concentration of medicagenic acid aglycon in the BJ varied between 2.4 and 2.8 ng/mL and 6.7–13.8 ng/mL in non-fermented and fermented BJ, respectively (Fig. 1). Our findings align with the data reported by^19^ regarding the concentration of medicagenic acid in non-fermented alfalfa BJ, which varied from 0.72 to 28.53 ng/mL.

**Table 1:** Comparison of identified anti-nutritional compounds of non-fermented and lacto-fermented BJ from fresh alfalfa biomass (*Medicago sativa* L.).

Compound	Formula	Non-Fermented BJ	Lacto-Fermented BJ	Rt	[M - H]^−^	Fragmens 1	Fragmens 2	Fragmens 3	Fragmens 4	Fragmens 5
Azukisaponin II	C_42_H_68_O_14_	+	-	38.67	795.45308	729.3805	113.0232			
Unknown saponin. Aglycon: 456.32396 (C_29_H_44_O_4_)	C_58_H_92_O_29_	+	-	32.02	1251.56460	1089.5095	949.4331	455.3185		
Unknown saponin. Aglycon: 504.34509 (C_30_H_48_O_6_)	C_48_H_76_O_21_	+	-	32.19	987.48009	925.4807	779.4272	661.3683	585.3482	503.3391
Unknown saponin. Aglycon: 504.34509 (C_30_H_48_O_6_)	C_48_H_76_O_21_	+	-	32.51	987.48009	925.4817	779.4212	661.3619	585.3405	503.3401
Unknown saponin. Aglycon: 504.34509 (C_30_H_48_O_6_)	C_41_H_64_O_16_	+	-	32.59	811.41161	749.4141	661.3580	503.3399	249.1108	
Unknown saponin. Aglycon: 504.34509 (C_30_H_48_O_6_)	C_42_H_66_O_17_			32.72	841.42218	797.4299	779.4215	661.3619	503.3387	455.3153
Unknown saponin. Aglycon: 440.32905 (C_29_H_44_O_3_)	C_58_H_92_O_28_	+	-	34.83	1235.56969	439.3222	221.0663			
Unknown saponin. Aglycon: 440.32905 (C_29_H_44_O_3_)	C_47_H_74_O_19_	+	-	35.10	941.47461	717.4210	439.3225			
Unknown saponin. Aglycon: 486.33452 (C_30_H_46_O_5_)	C_42_H_64_O_16_			36.64	823.41162	761.4146	643.3493	599.3600	567.3331	485.3280
Medinoside E or isomer	C_54_H_88_O_23_	+	-	32.34	1103.56381	205.0719	193.0350	113.0230		
Medinoside E or isomer	C_54_H_88_O_23_	+	-	32.63	1103.56381	205.0723	193.0349	113.0231		
Medicoside G (Medicagenic acid 3,28-di-O-glucoside)	C_42_H_66_O_16_	+	-	34.19	825.42727	645.3684	601.3768	569.3469	487.3440	439.3230
Medicoside J (Medicagenic acid 3-O-glucosyl-28-O-[xylosyl-(1→4)-rhamnosyl-(1→2)-arabinosyl]ester)	C_52_H_82_O_23_	+	-	35.05	1073.51687	663.3779	483.3115	439.3223		
Medicoside H (Medicagenic acid 3-O-glucosyl-28-O-[ rhamnosyl-(1→2)-arabinosyl]ester)	C_47_H_74_O_19_	+	-	34.07	941.47461	663.3724	645.3669	601.3795	487.3426	439.3231
Medicagenic acid 3-O-[galactosyl-(1→2)-glucoside]-28-O-glucosyl ester	C_48_H_76_O_21_	+	-	34.10	987.48009	879.4717	645.3649	601.3723	487.3418	439.3250
Medicagenic acid 3-O-glucuronide-28-O-[xylosyl-(1→4)-rhamnosyl-(1→2)-arabinosyl]ester	C_52_H_80_O_24_	+	-	34.67	1087.49613	911.4686	501.3233	439.3222	193.0349	113.0231
Medicagenic acid 28-O-[xylosyl-(1→4)-rhamnosyl-(1→2)-arabinosyl]ester	C_46_H_72_O_18_	+	-	36.50	911.46405	501.3231	483.3132	439.3220		
Medicagenic acid	C_30_H_46_O_6_	+	+	39.82	501.3231	483.3122	453.3013	437.3069	425.3063	409.2740

Lacto-fermentation significantly increased the concentration of medicagenic acid compared to non-fermented BJ, corroborating the findings of^21^, who observed a rise in medicagenic acid concentration with *Pediococcus* inoculation.

## Effect of lacto-fermentation on the concentration of phytohormones in alfalfa BJ

Twenty-three distinct derivatives of phytohormones were identified and quantified in alfalfa-derived BJ (Table 2). Since it lacks living cells due to its production process, the elevation in hormone levels post lacto-fermentation could be attributed to the activity of the bacterial inocula^22,23^. This elevation was observed for indole-3-acetic acid, active gibberellin GA1, jasmonic acid, abscisic acid, and salicylic acid. In the BJ, the concentration of salicylic acid was measured at 19 µg/mL. Salicylic acid has been recognized for its potential to promote plant growth and lutein biosynthesis^24^, particularly at lower concentrations^25^, while higher concentrations may exert inhibitory effects on the growth of certain plant species.

The reduction in various aromatic compounds (such as p-coumaric acid, caffeic acid, ferulic acid, and dihydroxy benzoic acids) is presumed to stem from their consumption by bacteria to synthesize diverse compounds with aromatic characteristics, including salicylic acid and auxins. The jasmonic acid isoleucine conjugate, recognized as the active form of jasmonic acid, originates from the plant. The concentration of jasmonic acid in BJ was found to be 290 µg/mL^21^ reported that the application of jasmonic acid within a range of 0.1 µg/mL to 45 mg/mL stimulated plant growth under various abiotic stresses, including cold, drought, salinity, heavy metals, light exposure, O3 exposure, imazapic, and circadian influences.

**Table 2:** Comparison of plant hormones of non-fermented (NF) and lacto-fermented (LF) BJ from fresh alfalfa biomass (*Medicago sativa* L.). Sample size (*n* = 3). Different letters following means in each column show significant differences at the level of *p* < 0.05.

Components		Non-Fermented BJ	Lacto-Fermented BJ
Higher abundant components µg BJ ml^**−1**^	salicylic acid	16.50 ± 0.00^b^	19.00 ± 0.12^a^
	2.5-dihydroxybenzoic acid	11.97 ± 0.55^a^	3.73 ± 0.25^b^
	2.6-dihydroxybenzoic acid	9.02 ± 0.08^a^	3.24 ± 0.04^b^
	benzoic acid	4.20 ± 0.12^b^	6.76 ± 0.40^a^
	4-Hydroxybenzoic acid - pHBA	2.94 ± 0.00^a^	2.90 ± 0.03^a^
Mid/low abundance components ng BJ ml^**−1**^	3-Hydroxybenzoic acid	296 ± 2.00^b^	342 ± 6.00^a^
	jasmonic acid	126.10 ± 1.30^b^	290 ± 2.00^a^
	sinapic acid	78.20 ± 0.60^b^	89.40 ± 1.00^a^
	abscisic acid	161.30 ± 1.10^b^	184.90 ± 2.90^a^
	phaseic acid	9.57 ± 0.01^b^	116.10 ± 0.00^a^
	dihydrophaseic acid	100.80 ± 1.40^a^	44.60 ± 1.70^b^
	3.4-dihydroxybenzoic acid	210 ± 0.00^a^	54.70 ± 2.50^b^
	Indole-3-acetic-Ac AUXIN	21.40 ± 0.40^b^	61 ± 1.00^a^
	p-coumaric acid	25.60 ± 0.40^a^	11.50 ± 0.76^b^
	caffeic acid	13.71 ± 0.51^a^	1.64 ± 0.06^b^
	ferulic acid	73.90 ± 1.50^a^	14.66 ± 1.72^b^
JA- conjug ng BJ ml^**−1**^	jasmonicAc-Isoleucine	29.70 ± 0.10^a^	10.71 ± 0.29^b^
	jasmonicAc-leucine	< 2^a^	< 2^a^
GAs ng BJ ml^**−1**^	GA_4_	< 2^a^	< 2^a^
	GA_20_	23.90 ± 3.50^a^	< 2^b^
	GA_3_	< 2^a^	< 2^a^
	GA_1_	9.71 ± 0.11^b^	13.21 ± 0.15^a^
	GA_8_	6.47 ± 0.07^a^	6.86 ± 0.30^a^
ISOTOPE QC recoveries 2H4 GA_1_ 2H2 GA_4_	ppb (ng/ml)	7.21 ± 0.01^b^	9.13 ± 0.13^a^
	Rec%	72.10 ± 0.10^b^	91.30 ± 1.30^a^
	ppb (ng/ml)	10.40 ± 0.32^b^	12.11 ± 0.07^a^
	Rec%	104 ± 3.20^b^	121.10 ± 0.70^a^

Lacto-fermentation also resulted in increased levels of GA8, considered an inactive gibberellin form, as well as benzoic acid, the precursor of salicylic acid, and phaseic acid, a byproduct of abscisic acid metabolism. The surplus is regarded as a metabolic byproduct of the microorganism. The concentration of GA3 in the BJ was measured at 2 ng/mL. Nonetheless, the application of GA3 at concentrations ranging from 150 to 400 mg/L has been shown to effectively enhance seed germination and seedling development under abiotic stress conditions^26,27^. Treating plants subjected to unfavorable growth conditions with abscisic acid at concentrations ranging between 161 and 184 ng/mL significantly improved plant growth. In our study, the concentration of abscisic acid in BJ ranged from 161 to 184 ng/mL. Normally, abscisic acid (ABA) is present at higher concentrations than phaseic acid (PA) in plant extracts depends on the plant species, developmental stage, and environmental conditions that information we can support by our results of BJ plant extract. According to our results, normal conditions (non-fermented BJ) shows different ratio 17:1 than literature (range of 2:1 to 10:1 (ABA: PA)), while under specific condition (fermentation) more ABA may be converted to phaseic acid leading to a shift in ratio. In our fermented BJ samples ABA: PA ratio was 1.6:1 in accordance to literature^28^.

According to the quantified plant hormones like SA and JA, BJ demonstrates potential for stimulating plant growth and development. Several identified phytohormones are present in concentrations conducive to improved plant growth, particularly under abiotic stress conditions. Trigonelline (N-methylnicotinamide) was detected by^29^ in samples of alfalfa BJ both before and after lacto-fermentation. This compound is known to play various regulatory roles in plant.

## Conclusions

The alfalfa-delivered BJ contains plant hormones, some of which are in outstanding abundance. Lacto-fermentation induced quantitative changes in the plant hormone compounds: an increase in the concentration of 10 plant hormones and a significant decrease in the concentration of mainly phenolic components were realized. The plant hormones in the BJ presented demonstrate one of the reasons for the effectiveness of the fermented brown juice (the presence of plant growth promoting bacteria (PGPB), the presence of organic acids, and the added nutritional value, in addition to the fact that lacto-fermentation stabilizes and makes the BJ shelf stable) as a plant biostimulant. The use of the alfalfa-delivered BJ as a nutrient medium for microorganism is appreciated because the bacteria that proliferate during lacto-fermentation have significantly increased the levels of plant hormones, thus is role in stimulation of plant growth is more enhanced. The alteration in medicagenic acid concentration can be attributed to the identification of at least seven medicagenic acid derivatives with sugar side chains in non-fermented alfalfa BJ, all of which were absent in fermented alfalfa BJ. Therefore, it is probable that only the relative proportion of medicagenic acid aglycone increased, rather than its absolute concentration. Although cytokinins and triacontanol (TRIA), plant growth regulators found in alfalfa, were not assessed in this study, future research will focus on exploring these components.

## Methods

### Origin of BJ

The alfalfa-derived BJ was graciously provided by Tedej Ltd. Company (Hajdúnánás, Hungary) subsequent to the mechanical pressing of alfalfa fresh biomass into green juice and fiber fractions at the Proteomill Pilot Plantation, specifically designed for leaf protein isolation^5^. The BJ was separated from coagulated leaf proteins using a cloth filter. Fresh BJ was promptly stored at -80 ℃ for subsequent measurements due to its rapid spoilage at room temperature. Lacto-fermentation of the fresh BJ was identified as a significant process to enhance its stability at room temperature while simultaneously improving its nutrient composition. Three bacterial strains—*Pediococcus acidilactici*, *Lactobacillus paracasei*, and *Lactobacillus plantarum*—were utilized for lacto-fermentation at 35 ℃ for 48 h at a concentration of 10^11^ CFU (cell forming unit), following the method described by^4^.

### Screening and quantification of saponins by UHPLC-ESI-ORBITRAP-MS/MS

Brown juice samples underwent filtration using a 0.22 μm PTFE syringe filter. Qualitative analysis of saponins in fermented and non-fermented alfalfa-derived BJ was conducted using UHPLC-ESI-ORBITRAP-MS/MS (ultra-high performance liquid chromatography-electrospray ionization-Orbitrap mass spectrometry), following a method adapted from Kaszás et al.^30^. The analytical setup included a UHPLC system Dionex Ultimate 3000RS/Thermo Fisher, Waltham, MA, USA, coupled with a Thermo Q Exactive Orbitrap hybrid mass spectrometer. Separation was achieved using a Thermo Accucore C18 analytical column (100/2.1 mm, 2.6 μm particle size) with gradient elution. Quantitative analysis employed medicagenic acid (≥ 99%) as an external standard (Merck-Sigma, Darmstadt, Germany). MS data were collected in both positive (4.0 kV electrospray voltage) and negative (3.8 kV electrospray voltage) ion modes in different runs using. The following settings were used for MS analyses: resolution: 70,000 in the cases of full scans and 35,000 in the cases of fragmentation scans; collision energy: 30 NCE; scan range: 100 to 1500 *m*/*z.* Trace Finder 3.1 (Thermo Scientific) software was used to analyse the raw files. The secondary metabolites were identified on the basis of our previous published works and our and online databases (Metlin, Massbank of North America, mzCloud). In every case, the exact molecular mass, isotopic pattern, characteristic fragment ions, and retention time were used for the identification of the secondary metabolites. The difference between the measured and calculated monoisotopic molecular masses was less than 5 ppm in every case.

## Targeted plant hormone analysis by ultra-performance liquid chromatography–tandem mass spectrometry (UPLC-MS/MS)

Upon thawing the frozen BJ and homogenizing via vortex mixing, 0.5 mL was combined with 5 ng of [2H4] GA 1 and [2H2] GA 4 as internal standards (OlChemIm s.r.o., Olomouc, Czech Republic) and diluted with 1.5 mL methanol (UPLC gradient grade; VWR, Radnor, PA, USA) in 2 mL safety Eppendorf tubes. Following vortexing, samples underwent centrifugation at 16,000 g and 4 °C for 15 min and then filtration through 0.22 μm PTFE syringe filters. The filtered samples were frozen at -20 ℃ overnight, followed by centrifugation upon thawing and vortexing at 16,000 g and 4 °C for 15 min, and filtration again through 0.22 μm PTFE syringe filters. Subsequently, samples underwent flow-through type SPE clean-up, where a Biotage ENVI C18 SPE tube (1 mL; Biotage; Uppsala, Sweden) was initially conditioned with 2.0 mL methanol and equilibrated with 2.0 mL 75% (v/v) methanol. Samples were loaded and flowed through in a 15 mL centrifuge tube. The SPE cartridge was then washed with 1.0 mL 80% (v/v) methanol, with this washing solution introduced into the same centrifuge tube. The final (approximately 3 mL) sample extract was evaporated under vacuum at 35 ℃, reconstituted in 1 mL of 30 v/v% methanol containing 0.1 v/v% formic acid (MS grade; VWR), filtered through a 0.22 μm pore-sized disposable PTFE syringe filter, and immediately submitted for analysis into the UPLC-US-MS/MS set-up at a final sample ratio of 0.5 mL/mL. Following UPLC separation, tandem mass spectrometric detection was carried out as follows also described by^31^. For UPLC separation a Waters Acquity I class UPLC system (Milford, MA, USA) was used and separation was achieved on a Waters Acquity HSS T3 column (1.8 μm, 100 mm × 2.1 mm), kept at 40 °C. Mobile phase A was water containing 0.1 v/v % formic acid (FA), while mobile phase B was acetonitrile (UPLC-MS grade; VWR) containing 0.1 v/v % FA. The flow was 0.4 mL/min, and the gradient profile was as follows: 0 min, 5% B; from 0 to 3 min, linear gradient to 20% B; from 3 to 4.3 min, isocratic 20% B; from 4.3 to 9 min, linear gradient to 45% B; from 9 to 11 min, linear gradient to 100% B; from 11 to 13 min, kept at 100% B; from 13.01 to 15 min, back to the initial conditions of 5% B. The injection volume was 1 µl for all samples that were kept at 8 °C in the auto sampler during the analysis.

Tandem mass spectrometric detection was performed on a Waters Xevo TQ-XS equipped with a UniSpray™ source (US) operated in timed multiple reaction monitoring (MRM) mode with the following settings: impactor voltage was 2.2 kV in both positive and negative modes; nebulizer gas, 6.2 bar; desolvation temperature, 600 °C; cone gas flow, 450 L/h; desolvation gas flow, 1100 L/h. For collision gas argon (5.0 purity) was used with a gas flow of 0.15 ml/min. Unit resolution was applied to each quadrupole. Dwell time set to be automatically calculated to take at least twenty points across each peak for quantitation. Where possible, at least three MRM transitions were used for data acquisition and the transition having the highest S/N ratio was used for quantitation. Data processing was done using Waters MassLynx 4.2 and TargetLynx software. Calibration for target analytes were carried out against procedural solvent calibration of a reference mix solution. Besides the internal standard QC samples spiked with this mixture were also used to verify method performance at each batch to monitor recovery rates of compounds, which were obtained from OlChemim s.r.o., or Merck-Sigma group (Darmmstadt, Germany).

### Statistical analysis

Phytochemical screening and quantification in BJ were performed via HPLC-MS/MS. Phytochemical screening was carried out in one repetition, while the quantification and the phytohormone analysis was conducted with three repetitions using ultra-performance liquid chromatography-tandem mass spectrometry (UPLC-MS/MS). The obtained results underwent one-way ANOVA using SPSS Statistics software version 24. The experiments revealed a significant interaction between the two independent variables (fermented and non-fermented brown juice), and mean comparisons were conducted using Dunn’s Test at a significance level of *p* < 0.05.

## Data Availability

All data are available within the text.

## References

[1] Hadidi, M., Orellana Palacios, J. C., McClements, D. J., Mahfouzi, M. & Moreno, A. Alfalfa as a sustainable source of plant-based food proteins. *Trends Food Sci. Technol.* **135**, 202–214 (2023).

[2] Kaszás, L. et al. Refining high-quality leaf protein and valuable co-products from green biomass of Jerusalem artichoke (Helianthus tuberosus L.) for sustainable protein supply. *Biomass Conv Bioref*. **12**, 2149–2164 (2022).

[3] Domokos-Szabolcsy, É. et al. Green Biomass-Based protein for sustainable feed and food supply: an overview of current and future prospective. *Life* **13**, 307 (2023). 36836666 10.3390/life13020307 PMC9966994

[4] Bákonyi, N. et al. Chemical traits of fermented alfalfa brown juice: its implications on physiological, biochemical, anatomical, and growth parameters of Celosia. *Agronomy* **10**, 247 (2020).

[5] Fári, M. G. & Domokos-Szabolcsy, É. Method for Producing Plant Protein Coagulum. (2022).

[6] Domokos-Szabolcsy, É. et al. Comparison of wet fractionation methods for processing broccoli agricultural wastes and evaluation of the Nutri-Chemical values of obtained products. *Foods* **11**, 2418 (2022). 36010418 10.3390/foods11162418 PMC9407407

[7] Thomsen, M. H., Bech, D. & Kiel, P. Manufacturing of stabilised brown juice for L-lysine production-from university lab scale over pilot scale to industrial production. *Chem. Biochem. Eng. Q.* **18**, 37–46 (2004).

[8] Kisvarga, S. et al. Fermented alfalfa brown juice significantly stimulates the growth and development of sweet Basil (Ocimum Basilicum L.) plants. *Agronomy* **10**, 657 (2020).

[9] Makleit, P. Total saponin content (TSC) of different alfalfa (Medicago sativa L.) cultivars cultivated in field experiment. *Review Agric. Rural Development* (2021).

[10] Sen, S., Makkar, H. P. S. & Becker, K. Alfalfa saponins and their implication in animal nutrition. *J. Agric. Food Chem.* **46**, 131–140 (1998). 10554208 10.1021/jf970389i

[11] Sadowska, B. et al. New Pharmacological properties of Medicago sativa and Saponaria officinalis saponin-rich fractions addressed to Candida albicans. *J. Med. Microbiol.* **63**, 1076–1086 (2014). 24850879 10.1099/jmm.0.075291-0

[12] Shah, M. S., Supriya, D., Mayuri, G. & Oswal, D. R. J. A systematic review on one of the nutraceutical potential plant Medicago Sativa (Alfalfa). *World J. Pharm. Res.* **9**, 683–700 (2020).

[13] Fan, W. N., Yang, Y. X., Shi, Y. Q., Wang, C. Z. & Xu, B. Effects of Dietary Alfalfa Saponin on Digestive Physiology in Weaned Piglets. *IJAR* (2023). 10.18805/IJAR.BF-1686

[14] Rafińska, K., Pomastowski, P., Wrona, O., Górecki, R. & Buszewski, B. Medicago sativa as a source of secondary metabolites for agriculture and pharmaceutical industry. *Phytochem. Lett.* **20**, 520–539 (2017).

[15] Ismail, B. P., Senaratne-Lenagala, L., Stube, A. & Brackenridge, A. Protein demand: review of plant and animal proteins used in alternative protein product development and production. *Anim. Front.* **10**, 53–63 (2020). 33391860 10.1093/af/vfaa040 PMC7759735

[16] Rahate, K. A., Madhumita, M. & Prabhakar, P. K. Nutritional composition, anti-nutritional factors, pretreatments-cum-processing impact and food formulation potential of faba bean (Vicia faba L.): A comprehensive review. *LWT* **138**, 110796 (2021).

[17] Das, G., Sharma, A. & Sarkar, P. K. Conventional and emerging processing techniques for the post-harvest reduction of antinutrients in edible legumes. *Appl. Food Res.* **2**, 100112 (2022).

[18] Ciesarová, Z., Mikušová, L., Magala, M., Kohajdová, Z. & Karovičová, J. Chapter 17 - Nonwheat Cereal-Fermented-Derived products. In *Fermented Foods in Health and Disease Prevention* (eds Frias, J. et al.) 417–432 (Academic, 2017). 10.1016/B978-0-12-802309-9.00017-0.

[19] Kovács, Z. et al. Nutrichemical alterations in different fractions of multiple-harvest alfalfa (Medicago sativa L.) green biomass fortified with various selenium forms. *Plant. Soil.* **487**, 173–195 (2023).

[20] Bialy, Z., Jurzysta, M., Oleszek, W., Piacente, S. & Pizza, C. Saponins in alfalfa (*Medicago sativa* L.) root and their structural Elucidation. *J. Agric. Food Chem.* **47**, 3185–3192 (1999). 10552628 10.1021/jf9901237

[21] Wang, B., Gao, R., Wu, Z. & Yu, Z. Functional analysis of sugars in modulating bacterial communities and metabolomics profiles of Medicago sativa silage. *Front Microbiol* **11**, (2020). 10.3389/fmicb.2020.00641 PMC7232540 32477276

[22] Spaepen, S. Plant hormones produced by microbes. In *Principles of Plant-Microbe Interactions: Microbes for Sustainable Agriculture* (ed. Lugtenberg, B.) 247–256 (Springer International Publishing, 2015). 10.1007/978-3-319-08575-3_26.

[23] Arshad, M. & Frankenberger, W. T. Microbial production of plant hormones. *Plant. Soil.* **133**, 1–8 (1991).

[24] Ahmed, N. R. et al. Exploring biostimulation of plant hormones and nitrate supplement to effectively enhance biomass growth and lutein production with thermo-tolerant *Desmodesmus* Sp. F51. *Bioresour. Technol.* **291**, 121883 (2019). 31387052 10.1016/j.biortech.2019.121883

[25] Li, A., Sun, X. & Liu, L. Action of Salicylic acid on plant growth. *Front Plant. Sci* **13**, (2022). 10.3389/fpls.2022.878076 PMC9093677 35574112

[26] Abdullah, B. S. & Abdulrahman, Y. A. Effect of different concentrations of gibberellic acid on seeds germination and growth in different turf grass genera. *Kufa J. Agricultural Sciences* **9**, (2017).

[27] Chauhan, A. et al. Influence of gibberellic acid and different salt concentrations on germination percentage and physiological parameters of oat cultivars. *Saudi J. Biol. Sci.* **26**, 1298–1304 (2019). 31516361 10.1016/j.sjbs.2019.04.014 PMC6733775

[28] Nambara, E. & Marion-Poll, A. Abscisic acid biosynthesis and catabolism. *Annu. Rev. Plant. Biol.* **56**, 165–185 (2005). 15862093 10.1146/annurev.arplant.56.032604.144046

[29] Barna, D. et al. Bioactive metabolite profile and antioxidant properties of brown juice, a processed alfalfa (Medicago sativa) by-product. *Heliyon* **8**, e11655 (2022). 36444258 10.1016/j.heliyon.2022.e11655 PMC9699961

[30] Kaszás, L. et al. Identification of bioactive phytochemicals in leaf protein concentrate of Jerusalem artichoke (Helianthus tuberosus L). *Plants (Basel)*. **9**, 889 (2020). 32674454 10.3390/plants9070889 PMC7411585

[31] Pál, M. et al. Light spectral composition modifies polyamine metabolism in young wheat plants. *Int. J. Mol. Sci.* **23**, 8394 (2022). 35955528 10.3390/ijms23158394 PMC9369354

